# The magnitude of exercise‐induced progenitor cell mobilisation and extravasation is positively associated with cardiorespiratory fitness

**DOI:** 10.1113/EP092041

**Published:** 2024-10-30

**Authors:** Mark Ross, Sarah Aldred, Mark T. Drayson, Jos A. Bosch, James E. Turner

**Affiliations:** ^1^ Institue of Life and Earth Sciences Heriot‐Watt University Edinburgh UK; ^2^ School of Sport, Exercise and Rehabilitation Sciences University of Birmingham Birmingham UK; ^3^ Institute of Immunology and Immunotherapy University of Birmingham Birmingham UK; ^4^ Department of Clinical Psychology University of Amsterdam Amsterdam The Netherlands

**Keywords:** cardiorespiratory fitness, exercise, progenitor cells

## Abstract

CD34^+^ progenitor cells with angiogenic capabilities traffic into blood during exercise and extravasate afterwards but the magnitude of this response varies between people. We examined whether exercise‐induced progenitor cell trafficking is influenced by cardiorespiratory fitness (maximum oxygen uptake; ${\dot{V}}_{O_{2}\max}$). Ten males (age: 23 ± 3 years; ${\dot{V}}_{O_{2}\max}$: 61.88 ± 4.68 mL kg min^−1^) undertook 1 h of treadmill running at 80% of ${\dot{V}}_{O_{2}\max}$. Blood samples were collected before exercise (Pre), in the final minute of exercise (0 h) and afterwards at 0.25, 1 and 24 h. Pan‐progenitor cells (CD34^+^, CD34^+^CD45^dim^) and putative endothelial progenitor cells (CD34^+^CD133^+^, CD34^+^VEGFR2^+^, CD34^+^CD45^dim^VEGFR2^+^) were quantified using flow cytometry. Progenitor subpopulations (except for CD34^+^CD45^dim^VEGFR2^+^) increased at 0 h (*P <* 0.05) and returned to pre‐exercise levels by 1 h. ${\dot{V}}_{O_{2}\max}$ was positively associated with the exercise‐induced progenitor cell response and there were statistically significant time × ${\dot{V}}_{O_{2}\max}$ interactions for CD34^+^, CD34^+^CD45^dim^ and CD34+CD133^+^ subpopulations but not VEGFR2‐expressing progenitor cells. There were statistically significant correlations between ${\dot{V}}_{O_{2}\max}$ and *ingress* (*r *> 0.70, *P *< 0.025) and *egress* (*r *> −0.77, *P *< 0.009) of progenitor cell subsets (CD34^+^, CD34^+^CD45^dim^, CD34^+^CD133^+^), showing that cardiorespiratory fitness influences the magnitude of progenitor cell mobilisation into the blood and subsequent extravasation. These data may provide a link between high levels of cardiorespiratory fitness and vascular health.

## Abstract (highlights)

- **What is the central question of the study?**
  Is the magnitude of progenitor cell trafficking into and out of the blood with a vigorous bout of exercise influenced by cardiorespiratory fitness?
- **What is the main finding and its importance?**
  Acute vigorous exercise bouts mobilise pan‐progenitor cells and their sub‐populations into blood, including endothelial progenitor cells, which subsequently extravasate to peripheral tissues post‐exercise. The magnitude of this response is strongly associated with cardiorespiratory fitness. Importantly, this finding identifies possible mechanisms underlying associations between an active lifestyle and endothelial health.

## 1 INTRODUCTION

Subpopulations of CD34^+^ pan‐progenitor cells—in particular, putative endothelial progenitor cells (EPCs)—might maintain vascular homeostasis by promoting endothelial regeneration, most likely via paracrine mechanisms, such as by secreting vascular endothelial growth factor (VEGF) (Hur et al., 2004). The frequency of putative EPCs in peripheral blood is an independent predictor of endothelial function (Sibal et al., 2009) despite these bone marrow‐derived cells representing a very small proportion of circulating cells (<0.02% of all peripheral blood mononuclear cells (PBMCs)) (Ross et al., 2018). People with cardiovascular disease or other vascular‐related diseases exhibit lower numbers of putative EPCs than age‐matched healthy controls (Fadini et al., 2005; Shantsila et al., 2012). In addition, low numbers of these cells can predict the occurrence of a significant cardiovascular event (Schmidt‐Lucke et al., 2005), or even premature mortality (Muggeridge et al., 2021).

Bouts of exercise transiently increase progenitor cell counts (Ross et al., 2014). However, resting and exercise‐induced progenitor cell counts show considerable inter‐individual variation, which is associated with age (Ross et al., 2018) and body mass index (BMI) (MacEneaney et al., 2009). In light of the established roles of exercise in progenitor cell mobilisation, and cardiorespiratory fitness in vascular health, it is conceivable that cardiorespiratory fitness may also contribute to the magnitude of progenitor cell responses to exercise, but this hypothesis has not yet been tested. The present study examined whether the magnitude of exercise‐induced progenitor cell and putative EPC trafficking is influenced by cardiorespiratory fitness. It was hypothesised that the largest exercise‐induced responses would be exhibited by the fittest individuals.

## 2 METHODS

### 2.1 Ethical approval

Ethical approval was granted by the School of Sport, Exercise and Rehabilitation Sciences research ethics committee of the University of Birmingham (reference 08/59). Participants provided written informed consent, and the study conformed to the standards set out by the *Declaration of Helsinki* (2004).

### 2.2 Participants

Ten healthy recreationally active males (age: 23 ± 3 years; height: 178.5 ± 5.5 cm; body mass: 72.5 ± 6.8 kg; BMI: 22.8 ± 1.9 kg m^2^; ${\dot{V}}_{O_{2}\max}$ 61.88 ± 4.68 mL kg min^−1^) took part. Participants self‐reported to be free from cardiovascular disease, type 2 diabetes and autoimmune inflammatory diseases, and were normotensive (blood pressure < 140/90 mmHg).

### 2.3 Assessment of maximal oxygen uptake

Maximal oxygen uptake (${\dot{V}}_{O_{2}\max}$) was measured during an incremental running test to volitional exhaustion (Turner et al., 2010, 2011). Breath‐by‐breath measurements were recorded every 5 s (Oxygcon Pro, Erich Jaeger GmbH, Hoechberg, Germany). Heart rate (HR) was measured using telemetry (RS200, Polar, Kempele, Finland) and ratings of perceived exertion (RPE) were recorded during the final minute of each stage.

### 2.4 Exercise trial

Participants ran for 1 h on a treadmill at speed to elicit 80% of ${\dot{V}}_{O_{2}\max}$, determined by assessing the relationship between oxygen uptake (${\dot{V}}_{O_{2}}$) and four submaximal running speeds in a previous test (Turner et al., 2010). Breath‐by‐breath measurements, HR and RPE were recorded every 5 min.

### 2.5 Blood sampling

Venous blood samples were collected before (Pre), in the final minute of exercise (0 h) and afterwards at 0.25, 1 and 24 h. Participants rested in a seated position for 1 h post‐exercise. In the 24 h prior to the trial, participants fasted from 22.00 h, did not consume alcohol or caffeine and did not exercise. In the 24 h post‐exercise, participants were free to go about their normal lifestyle. Exercise was avoided but light walking was permitted for transport purposes. Diet was not controlled, but participants fasted from 22.00 h prior to the 24 h blood sample and did not consume alcohol and caffeine.

### 2.6 Blood processing

Blood was collected in EDTA vacutainer tubes (BD Biosciences, Wokingham, UK) and processed within 3 h. Samples were lysed (1 mL whole blood: 9 mL of ammonium chloride‐based lysis buffer) and incubated for 10 min on ice. Samples were washed using phosphate buffered saline (PBS) by centrifuging at 250 *g* for 7 min and removing the supernatant before resuspending cells in 150 µL PBS. Cell preparations were incubated with CD34‐FITC (BD Biosciences, cat. no. 345801, clone: 8G12, dilution: 1:20), CD45‐PerCP (BD Biosciences, cat. no.: 345809, clone: 2D1, dilution: 1:10), VEGFR2‐APC (R&D Systems, Minneapolis, MN, USA, cat. no.: FAB357A, clone: 89106, dilution: 1:30) CD133‐PE (Miltenyi Biotech, Bergisch Gladbach, Germany, cat. no.: 130‐080‐801, clone: AC133, dilution: 1:20) for 20 min in the dark at room temperature and subsequently washed in PBS by centrifuging at 250 *g* for 7 min. Cell preparations were resuspended in PBS (2% paraformaldehyde) and stored in the dark at 4°C until analysis.

### 2.7 Flow cytometry

**FIGURE 1:**
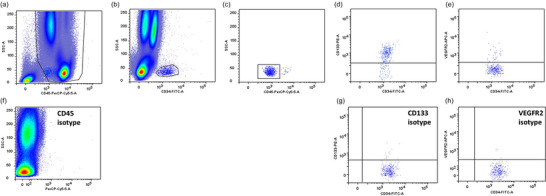
Flow cytometric quantification of progenitor cell subsets. (a) CD45^+^ leukocytes. (b) Gating of CD34^+^ (c) and CD34^+^CD45^dim^ pan‐progenitor cells. (d) CD34^+^CD133^+^ (e) and CD34^+^VEGFR2^+^ endothelial progenitor cells. CD34^+^CD45^dim^VEGFR2^+^ endothelial progenitor cells are not shown given the similarity in gating/plot to (e). Isotype controls for CD45 (f), CD133 (g) and VEGFR2 (h).

Cells were analysed within 24 h using a six‐colour flow cytometer (BD FACS CANTO II, BD Biosciences). Isotype controls were used to identify positive and negative populations where appropriate (Figure 1). Electronic compensation was applied prior to analysis. A minimum of 1,000,000 events were recorded per sample. Data were analysed using FlowLogic, Inivai Technologies, Mentone, Victoria, Australia). The putative EPC panel included markers of stemness (CD34, CD133) and an endothelial lineage marker (VEGFR2) (Fadini et al., 2008). Previous work highlights that CD45 expression can distinguish between early and late EPCs (Hur et al., 2004), which exhibit a different functional capacity to stimulate endothelial repair, with early EPCs acting in a paracrine manner by secreting pro‐angiogenic cytokines, and late EPCs having a greater endothelial differentiation potential (Hur et al., 2004). CD45^+^ cells were identified and electronically gated. Subsequently, CD45^+^ cells co‐expressing CD34 and with a low side scatter profile were identified as CD34^+^ progenitor cells. Expression of CD133 and VEGFR2 was analysed and CD34^+^ and CD34^+^VEGFR2^+^ cells that were dimly expressing CD45 were enumerated (Figure 1). Absolute cell counts (cells mL^−1^) were computed using a dual platform approach, by combining percentage values for gated cell populations with data from the leukocyte differential assessed on the same day and in the same blood sample (Coulter ACT^diff^; Beckman‐Coulter, High Wycombe, UK) correcting for blood volume changes in response to exercise (Dill & Costill, 1974).

### 2.8 Statistical analysis

Data were analysed with repeated measures analysis of variance (ANOVA) with *post hoc* Bonferroni tests where appropriate to determine differences between blood sampling time points. ANCOVA was used to examine the influence of ${\dot{V}}_{O_{2}\max}$ and partial eta squared (η_p_^2^) values were calculated for effect size. Pairwise comparisons were only conducted where a statistically significant main effect of time was observed. Pearson correlations determined the relationship between ${\dot{V}}_{O_{2}\max}$ (mL kg min^−1^) and progenitor cell *ingress* (Δ in absolute cell counts between Pre and 0 h post‐exercise) and *egress* (Δ in absolute cell counts between 0 and 1 h post‐exercise) with *r‐* and *P*‐values presented. Data were analysed using SPSS Statistics for Windows version 23 (IBM Corp., Armonk, NY, USA) with figures produced in GraphPad Prism version 9.4.1 (GraphPad Software, Boston, MA, USA). Statistical significance was considered to be *P* < 0.05.

## 3 RESULTS

### 3.1 Characteristics of the exercise bout

All participants completed the exercise bout, running for 1 h at 78.6 ± 1.3% of ${\dot{V}}_{O_{2}\max}$. The average HR was 173 ± 7 bpm (90 ± 4% HR_max_), the respiratory exchange ratio was 0.89 ± 0.04 and the RPE was 14 ± 2.

### 3.2 Exercise‐induced progenitor cell kinetics

**FIGURE 2:**
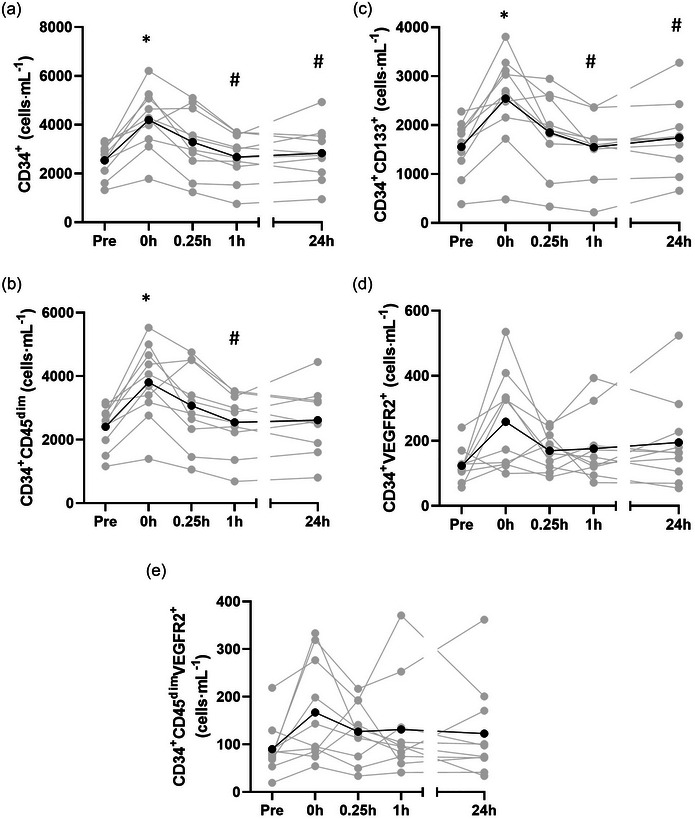
The effect of vigorous exercise on circulating progenitor cell subsets. (a) CD34^+^ and (b) CD34^+^CD45^dim^ pan‐progenitor cells. (c) CD34^+^CD133^+^ (d), CD34^+^VEGFR2^+^ and (e) CD34^+^CD45^dim^VEGFR2^+^ endothelial progenitor cells (*n* = 10). Values shown are means (black lines + circles) and individual values (grey lines + circles). *Significantly different from Pre; #significantly different from 0 h. (a) CD34: *P < 0.001; #0 to 1 h, *P* < 0.001, 0 to 24 h, *P* = 0.002; (b) CD34^+^CD45^dim^: *P = 0.007; #0 to 1 h, *P* = 0.008; 0 to 24 h, *P* = 0.019; (c) CD34^+^CD133^+^: *P = 0.006; #0 to 1 h, *P* = 0.012, 0 to 24 h, *P* = 0.002.

Table 1 shows that there were statistically significant main effects of time for pan‐progenitor cells, identified as either CD34^+^ (*P* < 0.001, η_p_^2 ^= 0.631) or CD34^+^CD45^dim^ (*P* < 0.001, η_p_^2 ^= 0.584) and putative EPCs, identified as CD34^+^CD133^+^ (*P* < 0.001, η_p_^2 ^= 0.581) or CD34^+^VEGFR2^+^ (*P* = 0.016, η_p_^2 ^= 0.281). These cells showed a statistically significant increase from pre‐exercise to 0 h (*P <* 0.001) except for CD34^+^VEGFR2^+^ (*P = *0.240) (Table 1, Figure 2). There was not a statistically significant main effect of time when EPCs were identified as being CD34^+^CD45^dim^VEGFR2^+^ (*P* = 0.083, η_p_^2 ^= 0.200) (Table 1, Figure 2). All cells remained at pre‐exercise levels 1 and 24 h post‐exercise (Table 1, Figure 2).

**TABLE 1:** The effects of cardiorespiratory fitness on exercise‐induced progenitor cell kinetics (*n* = 10).

	Pre	0 h	0.25 h	1 h	24 h	Main effect of time (Model 1)	Adjusted main effect of time (Model 2)	Time × ${\dot{V}}_{O_{2}\max}$ interaction
**CD34^+^**	2540 ± 213	4197 ± 392*#	3287 ± 421	2675 ± 304#	2839 ± 353	*F* = 15.4, *P* < 0.001, η_p_^2 ^= 0.631	*F* = 3.1 *P* = 0.028, η_p_^2 ^= 0.281	*F =* 4.3, *P =* 0.007 η_p_^2 ^= 0.348
**CD34^+^CD45^dim^**	2409 ± 211	3806 ± 380*#	3073 ± 402	2547 ± 294#	2618 ± 324	*F* = 12.6, *P* < 0.001, η_p_^2 ^= 0.584	*F* = 3.3, *P* = 0.024, η_p_^2 ^= 0.289	*F =* 4.3, *P =* 0.007 η_p_^2 ^= 0.350
**CD34^+^CD133^+^**	1555 ± 182	2540 ± 259*#	1849 ± 253	1547 ± 200#	1741 ± 233	*F* = 12.5, *P* < 0.001, η_p_^2 ^= 0.581	*F* = 4.0, *P* = 0.010, η_p_^2 ^= 0.332	*F =* 5.2, *P =* 0.010 η_p_^2 ^= 0.396
**CD34^+^VEGFR2^+^**	124 ± 17	259 ± 48	170 ± 19*	176 ± 32	195 ± 39	*F* = 3.5, *P* = 0.020, η_p_^2 ^= 0.281	*F* = 1.4, *P* = 0.271, η_p_^2 ^= 0.145	*F =* 1.6, *P =* 0.194, η_p_^2 ^= 0.168
**CD34^+^CD45^dim^VEGFR2^+^**	90 ± 17	167 ± 34	127 ± 20	132 ± 32	123 ± 31	*F* = 2.253, *P* = 0.083, η_p_^2 ^= 0.200	*F* = 0.9, *P* = 0.504, η_p_^2 ^= 0.096	*F =* 0.9, *P =* 0.478 η_p_^2 ^= 0.101

*Note*: Values shown are means ± SD. *P < 0.001 vs. pre‐exercise. Model 1 = unadjusted ANOVA, Model 2 = ANCOVA with ${\dot{V}}_{O_{2}\max}$ entered as a covariate. #Statistically significant time × ${\dot{V}}_{O_{2}\max}$ interaction effect for ingress (change from pre to 0 h) or *egress* (change from 0 to 1 h). ${\dot{V}}_{O_{2}\max}$, maximum oxygen uptake.

### 3.3 Effects of cardiorespiratory fitness on exercise‐induced progenitor cell kinetics

Table 1 shows that the statistically significant main effects of time for exercise‐mobilised progenitor cell populations remained when ${\dot{V}}_{O_{2}\max}$ was controlled for among CD34^+^ (*P* = 0.028, η_p_^2 ^= 0.281), CD34^+^CD45^dim^ (*P* = 0.024, η_p_^2 ^= 0.289) cells and CD34^+^CD133^+^ putative EPCs (*P* = 0.010, η_p_^2 ^= 0.332). There were statistically significant time × ${\dot{V}}_{O_{2}\max}$ interaction effects for these cell subsets (CD34^+^: *P* = 0.007, η_p_^2 ^= 0.348; CD34^+^CD45^dim^: *P* = 0.007, η_p_^2 ^= 0.350; CD34^+^CD133^+^: *P* = 0.001, η_p_^2 ^= 0.396). There were no statistically significant time × ${\dot{V}}_{O_{2}\max}$ interaction effects for CD34^+^VEGFR2^+^ (*P* = 0.194, η_p_^2 ^= 0.168) or CD34^+^CD45^dim^VEGFR2^+^ subsets (*P* = 0.478, η_p_^2 ^= 0.101).

As exercise stimulates the trafficking of pan‐progenitor and EPCs *into* the blood (*ingress*) and *out* of the blood (*egress*), we examined whether ${\dot{V}}_{O_{2}\max}$ influenced the change from pre‐ to 0 h (*ingress*) and the change from 0 to 1 h post‐exercise (*egress*) (Table 1). There were statistically significant time × ${\dot{V}}_{O_{2}\max}$ interaction effects for pan‐progenitor cell *ingress* among CD34^+^ (*P* = 0.002, η_p_^2 ^= 0.704), CD34^+^CD45^dim^ (*P* = 0.006, η_p_^2 ^= 0.625) and CD34^+^CD133^+^ putative EPCs (*P* = 0.010, η_p_^2 ^= 0.587), but no statistically significant interaction effects for CD34^+^VEGFR2^+^ (*P* = 0.145, η_p_^2 ^= 0.245) and CD34^+^CD45^dim^VEGFR2^+^ cells (*P* = 0.622, η_p_^2 ^= 0.032). Similarly, there were statistically significant time × ${\dot{V}}_{O_{2}\max}$ interaction effects for pan‐progenitor cell *egress* among CD34^+^ (*P* = 0.003, η_p_^2 ^= 0.508). CD34^+^CD45^dim^ (*P* = 0.005, η_p_^2 ^= 0.489) and CD34^+^CD133^+^ putative EPCs (*P* = 0.008, η_p_^2 ^= 0.451), but no statistically significant interaction effects for CD34^+^VEGFR2^+^ (*P* = 0.202, η_p_^2 ^= 181) and CD34^+^CD45^dim^VEGFR2^+^ cells (*P* = 0.264, η_p_^2 ^= 0.154).

**FIGURE 3:**
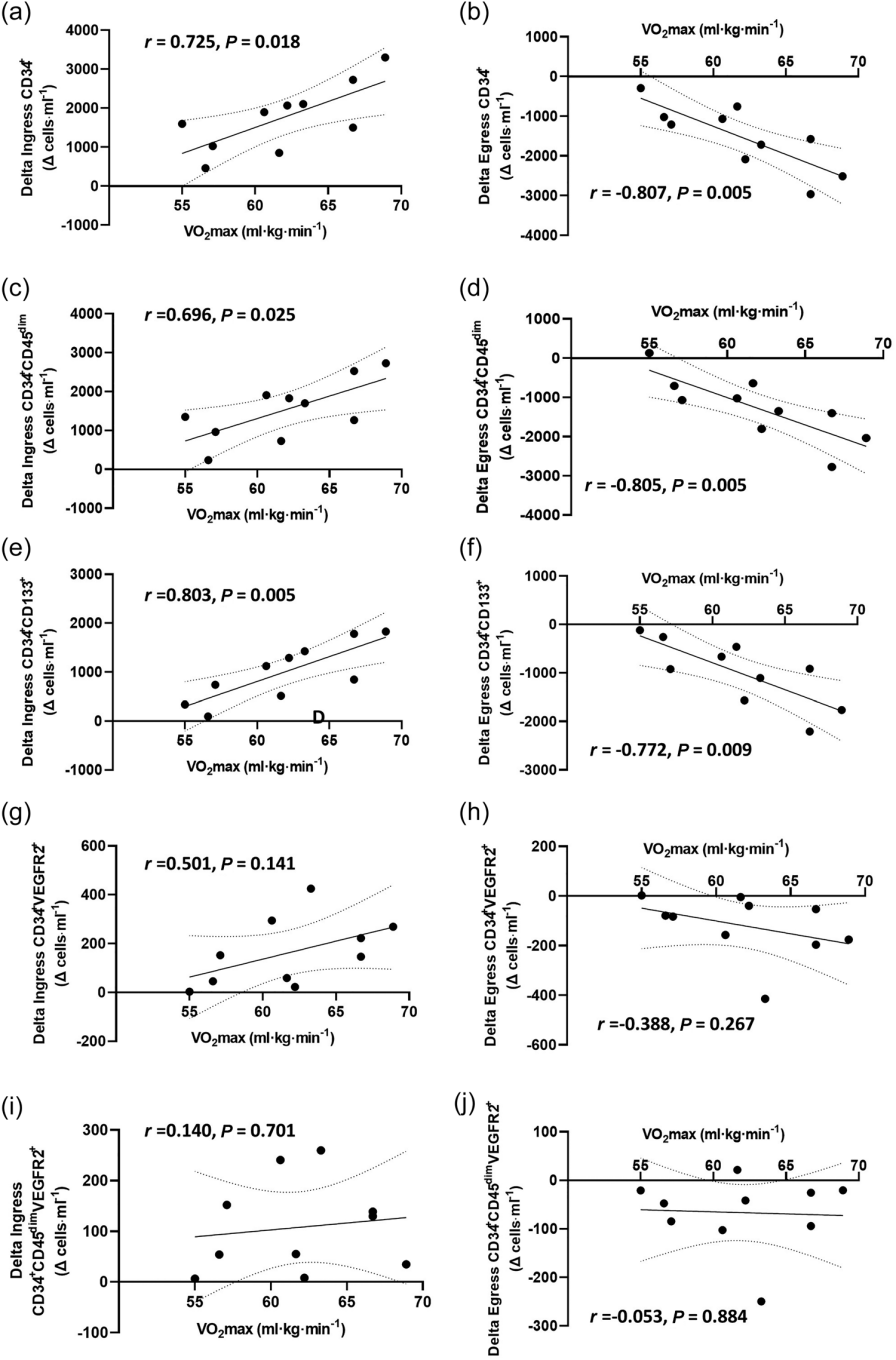
Pearson correlations between ${\dot{V}}_{O_{2}\max}$ and peak changes in exercise‐induced progenitor cell counts. Plots show ingress and egress of CD34^+^ (a, b), CD34^+^CD45^dim^ (c, d), CD34^+^CD133^+^ (e, f), CD34^+^VEGFR2^+^ (g, h), and CD34^+^CD45^dim^VEGFR2^+^ (i, j) cells (*n* = 10). *Ingress* represents a change in absolute cell counts between Pre and 0 h post‐exercise. *Egress* represents a change in absolute cell counts between 0 and 1 h post‐exercise.

In support, there were statistically significant positive correlations between peak *ingress* in cell counts from pre‐ to 0 h for pan‐progenitor cells (CD34^+^: *r* = 0.725, *P* = 0.018; CD34^+^CD45^dim^: *r* = 0.696, *P* = 0.025) and putative EPCs identified as CD34^+^CD133^+^ (*r* = 0.803, *P* = 0.005). There were no statistically significant correlations between ${\dot{V}}_{O_{2}\max}$ and *ingress* of EPCs identified as CD34^+^VEGFR2^+^ (*r* = 0.501, *P* = 0.141) or CD34^+^CD45^dim^VEGFR2^+^ (*r* = 0.140, *P* = 0.701) (Figure 3). Additionally, there were statistically significant negative correlations between peak *egress* of cell counts from 0 to 1 h post‐exercise for pan‐progenitor cells (CD34^+^: *r* = −0.807, *P* = 0.005; CD34^+^CD45^dim^: *r* = −0.805, *P* = 0.005) and EPCs identified as CD34^+^CD133^+^ (*r* = −0.772, *P* = 0.009). There were no statistically significant correlations between ${\dot{V}}_{O_{2}\max}$ and *egress of* CD34^+^VEGFR2^+^ (*r* = −0.388, *P* = 0.267) or CD34^+^CD45^dim^VEGFR2^+^ putative EPCs  (*r* = −0.053, *P* = 0.884) (Figure 3).

Finally, the statistically significant time × ${\dot{V}}_{O_{2}\max}$ interaction effects shown in Table 1 between pre‐ and 0 h (*ingress*) and 0 and 1 h post‐exercise (*egress*) were lost when subsequently controlling for other parameters using ANCOVA that reflect absolute exercise intensity (i.e., heart rate, oxygen uptake, running speed; data not shown).

## 4 DISCUSSION

This study showed that a bout of vigorous running exercise mobilises pan‐progenitor cells identified as CD34^+^ or CD34^+^CD45^dim^ and putative EPCs identified as CD34^+^CD133^+^, but not putative EPCs identified as CD34^+^VEGFR2^+^ or CD34^+^CD45^dim^VEGFR2^+^. In line with our hypothesis, this study additionally demonstrates that the magnitude of pan‐progenitor and putative endothelial progenitor cell *ingress* and *egress* is related to cardiorespiratory fitness.

We show for the first time that cardiorespiratory fitness is significantly correlated with the *ingress* (*r *> 0.700, *P *< 0.025) and *egress* (*r *> −0.770, *P *< 0.009) of pan‐progenitor cells and putative EPCs. However, despite carefully prescribing and maintaining exercise intensity (78.6 ± 1.3% of ${\dot{V}}_{O_{2}\max}$, 173 ± 7 bpm or 90 ± 4% maximum heart rate) and controlling statistically for ${\dot{V}}_{O_{2}\max}$, the magnitude of exercise‐induced progenitor cell kinetics was influenced by absolute exercise intensity (i.e., assessed via heart rate, ${\dot{V}}_{O_{2}}$ running speed). Thus, although ${\dot{V}}_{O_{2}\max}$ is associated with the magnitude of exercise‐induced pan‐progenitor cell and putative EPC *ingress* and *egress*, this is most likely because fitter individuals work at a higher absolute exercise intensity. Similar effects of exercise intensity have been reported previously (Niemiro et al., 2018). A plausible mechanism is that the release of chemoattractants into the circulation is exercise intensity‐dependent (Kindermann et al., 1982; Wahl et al., 2011). Thus, although cardiorespiratory fitness appears to influence the magnitude of exercise‐induced progenitor cell responses, this is most likely because fitter individuals work at a higher absolute intensity, and thus may gain the greatest health benefits from each exercise bout.

Our findings support previous studies, showing in general, that all forms of exercise mobilise pan‐progenitor cells into peripheral blood, including resistance exercise (Ross et al., 2014), short‐duration aerobic cycling (Ross et al., 2018) and maximal exercise tests (Van Craenenbroeck et al., 2008). These progenitor cells are presumably mobilised from the bone marrow, which is highly vascularised. Indeed, exercise‐induced stromal‐derived factor‐1 (SDF‐1) release is reported to act as a chemoattractant for CD34^+^ progenitor cells expressing the chemokine receptor C‐X‐C chemokine receptor 4 (CXCR4) (Chang et al., 2015). Other factors may stimulate progenitor cell mobilisation, such as VEGF, which acts via attracting VEGFR2^+^ cells across the bone marrow niche and into the circulation. In support, plasma VEGF is strongly associated with the mobilisation of CD34^+^ cells that occurs with myocardial infarction (Shintani et al., 2001). Further, VEGF is released from skeletal muscle in response to strenuous exercise (Hoier et al., 2010) and VEGF concentration in blood post‐exercise is positively correlated with change in progenitor cells (Adams et al., 2004). However, the time course of growth factor and chemokine release in response to exercise is not fully defined and may not align with the immediate mobilisation of progenitor cells. Other stimuli include nitric oxide (Aicher et al., 2003; Cubbon et al., 2010) but it is most established that catecholamines, such as adrenaline, are largely responsible for the immediate exercise‐induced CD34^+^ progenitor cell mobilisation which can be blocked by β‐antagonists (Agha et al., 2018). There may also be a role for exercise‐induced shear stress (due to elevated cardiac output and subsequent blood flow) causing the detachment of cells adhered to the endothelium (i.e., demargination). However, it has been shown that exercise‐induced progenitor cell mobilisation was unaffected by β_1_ selective antagonist administration which significantly reduced heart rate and blood pressure changes during exercise (Agha et al., 2018).

In our study, we did not observe any significant changes among CD34^+^VEGFR2^+^ or CD34^+^CD45^dim^VEGFR2^+^ cell subsets in response to exercise. It may be that exercise itself does not stimulate the mobilisation of these cells but given the large inter‐participant variability, our sample size is not large enough to reliably conclude exercise does not affect these cells. Indeed, in the present study, these cells are very low in number (approximately 1.95% of CD34^+^ cells for CD34^+^VEGFR2^+^ and approximately 0.67% of CD34^+^ cells for CD34^+^CD45^dim^VEGFR2^+^—and it is worth emphasising that CD34^+^ cells are approximately 6% of leukocytes among our participants). Moreover, we have reported statistically significant changes in CD34^+^, CD34^+^CD133^+^ but not among VEGFR2‐expressing progenitor cells elsewhere (Muggeridge et al., 2021).

A transient *ingress* followed by an *egress* of progenitor cells in response to exercise bouts is consistently reported (Mobius‐Winkler et al., 2009; Niemiro et al., 2017). The *egress* of progenitor cells could be due to a significant fall in circulating factors responsible for their presence in the blood (such as adrenaline and VEGF) but could also be due to active trafficking into ischaemic skeletal muscle (Palermo et al., 2005) as part of muscle and vascular remodelling. In support, Emmons et al. (2016) reported that skeletal muscle upregulates progenitor cell homing factors, such as stem cell factor, SDF‐1, angiopoietin‐1 (ANG‐1) and VEGF, and separately, it has been shown among wildtype mice that 1 week of exercise training increased the number of bone marrow‐derived cells within skeletal muscle (Palermo et al., 2005). It has also been suggested that putative EPCs traffic to extramedullary tissues post‐exercise, such as the spleen (Emmons et al., 2016), which also acts as a progenitor cell pool (Wolber et al., 2002). However, when interpreting exercise‐induced *ingress* and *egress* of cells, it should also be considered whether there is an overall change in the movement of cells *into* or *out of* the circulation, because even at rest, some cells will be entering blood, and other cells will be leaving. Thus, the net result of this homeostatic trafficking represents the ‘resting’ values of cells. In support, there is some evidence in lymphocytes that a decrease in the circulating pool (caused by glucorticosteroids), is caused by a *reduction* of cells *moving into* the circulation from lymphoid organs, rather than an *increase* of cells *leaving* blood, thus resulting in a lower ‘net’ circulating value, or apparent lymphocytopenia (Bloemena et al., 1990). Future studies are warranted to define the normal homeostatic circulation of progenitor cells under resting conditions using cell tracing methods.

An implication of this study is that exercise‐mobilised progenitor cells have potent vasculogenic potential (Chang et al., 2015), and these cells promote vascular endothelial repair in a variety of experimental models (Chang et al., 2015; Xia et al., 2012). Therefore, exercise‐induced progenitor cell mobilisation might be a possible mechanism for exercise‐induced angiogenesis and more broadly, maintenance of, or improvements in, vascular health (Ross et al., 2023; Yang et al., 2013). However, when interpreting the results of the present study, and speculating on the implications, it should be considered that the participants were regularly engaged in endurance exercise, as shown by their ${\dot{V}}_{O_{2}\max}$ of 61.88 ± 4.68 mL kg min^−1^ within the 99th percentile for age and sex (ACSM, 2022). Thus, further work should examine whether ${\dot{V}}_{O_{2}\max}$ also influences the magnitude of pan‐progenitor cell and putative EPC mobilisation in less active/fit individuals. In addition, this study only recruited males and future work should include female participants to examine if there are sex‐specific differences in progenitor cell number, function and mobilisation in response to stressors.

This study has shown that a bout of vigorous running causes a substantial movement of pan‐progenitor cells (identified as CD34^+^ or CD34^+^CD45^dim^) and putative EPCs (identified as CD34^+^CD133^+^, CD34^+^VEGFR2^+^ or CD34^+^CD45^dim^VEGFR2^+^) into and out of the circulation. The magnitude of these responses was greater among participants with higher cardiorespiratory fitness, which might imply that the links between a physically active lifestyle and vascular health could be mediated in part by EPC responses to exercise.

## AUTHOR CONTRIBUTIONS

Ross: Data curation, formal analysis, writing–original draft, writing–review & editing and visualisation. Aldred: Conceptualisation, writing–review & editing and funding acquisition. Drayson: Conceptualisation, methodology, resources, writing–review & editing and funding acquisition. Bosch: Conceptualisation, methodology, resources, writing–review & editing and funding acquisition. James Turner: Conceptualization, methodology, formal analysis, investigation, project administration, data curation, writing–original draft and writing–review & editing. All authors have read and approved the final version of this manuscript and agree to be accountable for all aspects of the work in ensuring that questions related to the accuracy or integrity of any part of the work are appropriately investigated and resolved. All persons designated as authors qualify for authorship, and all those who qualify for authorship are listed.

## CONFLICT OF INTEREST

None declared.

## Data Availability

Upon acceptance of this manuscript, data will be deposited open access and freely available via the University of Birmingham.
